# High-Density Tactile Sensor Array for Sub-Millimeter Texture Recognition

**DOI:** 10.3390/s25165078

**Published:** 2025-08-15

**Authors:** Chengran Cao, Guocheng Wang, Yixin Liu, Min Zhang

**Affiliations:** 1 Shenzhen International Graduate School, Tsinghua University, Shenzhen 518055, China; ccr22@tsinghua.org.cn (C.C.); wgc22@mails.tsinghua.edu.cn (G.W.); liu-yx19@mails.tsinghua.edu.cn (Y.L.); 2 PengCheng Laboratory, Shenzhen 518000, China

**Keywords:** tactile sensor, high-density array, texture recognition, microstructure, carbon nanotube

## Abstract

**Highlights:**

**What are the main findings?**

- Development of a high-density tactile sensor array featuring 10 μm pyramid microstructures and a CNT resistive layer, achieving ultra-high sensitivity (8.082 kPa^−1^) in the 0.2–0.5 kPa range and 500 μm spatial resolution—surpassing human fingertip discrimination thresholds.
- The sensor demonstrates exceptional performance; rapid response (125 ms loading), high stability (>1000 cycles), minimal unit variability (0.634% error), and reliable low-pressure detection (down to 90 Pa), validated through finite element simulations and experimental testing.

**What is the implication of the main finding?**

- Enables high-fidelity reconstruction of sub-millimeter textures (e.g., 500 μm patterns, Braille dots), advancing applications in prosthetics, robotics, and human–machine interfaces requiring ultra-fine tactile feedback.
- Establishes a scalable manufacturing paradigm using mold-based microstructure replication and solution-processable materials, overcoming traditional sensitivity-resolution trade-offs for future tactile sensing technologies.

**Abstract:**

High-density tactile sensor arrays that replicate human touch could restore texture perception in paralyzed individuals. However, conventional tactile sensor arrays face inherent trade-offs between spatial resolution, sensitivity, and crosstalk suppression due to microstructure size limitations and signal interference. To address this, we developed a tactile sensor featuring 10 μm-scale pyramid tips that achieve ultra-high sensitivity (8.082 kPa^−1^ in 0.2–0.5 kPa range). By integrating a flexible resistive sensing layer with a 256 × 256 active-matrix thin-film transistor (TFT) readout system, our design achieves 500 μm spatial resolution—surpassing human fingertip discrimination thresholds. The sensor demonstrates rapid response (125 ms), exceptional stability (>1000 cycles), and successful reconstruction of 500 μm textures and Braille patterns. This work establishes a scalable platform for high-fidelity tactile perception in static fine texture recognition.

## 1. Introduction

Tactile sensation serves as a fundamental interface for human interaction with the physical world, enabling the perception of critical surface properties such as texture and hardness [1,2,3]. This capability underpins essential functions including object manipulation and environmental awareness [4,5]. Crucially, human tactile perception operates within stringent spatiotemporal thresholds; for example, spatial resolution of ~1 mm at the fingertip, dynamic force detection range from 0.001 N to 10 N, and rapid signal processing with a latency of less than 50 ms [6,7]. These thresholds define the benchmark for artificial tactile systems aiming to replicate human texture recognition capabilities.

Current tactile sensors often struggle to achieve the sensitivity and resolution required for fine texture recognition. Many sensors can detect coarse textures but fail to distinguish subtle variations, such as the difference between fine silk and coarse cotton [8,9]. This limitation hinders their application in fields where precise texture discrimination is essential, such as robotics, prosthetics, and industrial automation [10].

Tactile sensing arrays refer to arrays of tactile sensors. While individual tactile sensors can achieve the conversion of physiological signals into electrical signals, they may lack the ability to recognize and display signals at each pixel. In contrast, tactile sensing arrays have the capability to display the detected pressure at a more granular level, providing a visual representation of pressure distribution [11,12,13,14,15,16]. This transformation enhances the perceptual understanding of pressure, turning it from an abstract concept into a tangible and visually interpretable representation through the array of sensors [17]. Tactile sensor arrays can elevate the resolution of the sensor array that concerns pressure signals by adjusting array density and pixel size. Drawing inspiration from the role of fingerprints in humans, which enhance the tactile sensitivity of Meissner corpuscles and contribute to the perception of intricate textures, the high-density sensor array strives to emulate this natural phenomenon [8,18,19]. The sensor array replicates the fingerprint patterns, the ridges and valleys of which are about 100 μm, through the fabrication of microstructures [20,21]. This emulation not only mirrors the inherent tactile sensitivity observed in humans but also significantly bolsters the sensor’s ability to discern and interpret delicate textures.

Significant advances have been made in developing sensor arrays for tactile mapping [22,23]. An et al. [1] proposed transparent fingerprint sensors that achieve multiplexed pressure detection but exhibit limited sensitivity of 1.78 × 10^−3^ kPa^−1^. Chun et al. [24] present interlocked ITO nanosprings that enable fabric classification with 99.8% accuracy but with a spatial resolution of ~196 pixels/cm^2^. Recent work has also explored triboelectric and capacitive approaches; Zhang et al. [25] developed a self-powered wireless capacitive triboelectric pressure sensor achieving 78.78 V kPa^−1^ sensitivity and 20 ms response time, while Zhou et al. [26] created a flexible triboelectric insole array for gait analysis with deep learning integration. While ZnO-constrained nanotube arrays on graphene offer individually addressable pixels [27], piezotronic transistors still face manufacturing scalability challenges [28]. Conformal printing strategies broaden detection ranges but compromise resolution [29]. Despite these advancements, a critical gap persists; high-density resistive sensor arrays struggle to simultaneously achieve high sensitivity and high spatial resolution in the low-pressure regime.

Here, we propose a bioinspired high-density tactile sensor array featuring pyramid microstructures that overcome the sensitivity-resolution trade-off. The sensor integrates a carbon nanotube (CNT)-based resistive sensing layer with a thin-film transistor (TFT) active matrix. The resulting device exhibits exceptional performance and reconstructs sub-millimeter textures. This work establishes a new paradigm for high-fidelity tactile sensing through pyramid microstructure optimization and scalable manufacturing, with transformative potential for robotics, prosthetics, and human–machine interfaces requiring ultra-fine tactile feedback.

## 2. Materials and Methods

### 2.1. Fabrication of the Sensor Array

**Figure 1:**
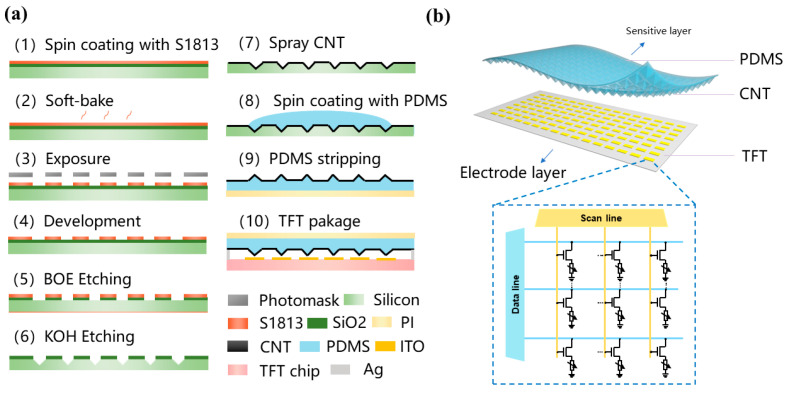
(**a**) Illustration of the sensor array. (**b**) Equivalent circuit diagram of the TFT array.

The fabrication process of the proposed sensor array is illustrated in Figure 1a. To create a resistive layer with fine microstructures within the sensor, it is first necessary to fabricate a mold featuring a micro-pyramid array structure. A 2-inch <100> crystalline silicon oxide wafer is used for mold fabrication. After uniformly coating the silicon wafer with photoresist S1813, a series of photolithographic steps are carried out, including soft baking, exposure, development, hard baking, and back-side protection, to obtain the desired pattern on the wafer. Buffered Oxide Etch (BOE) is then performed, followed by photoresist removal and anisotropic etching with potassium hydroxide (KOH). The BOE solution consists of hydrofluoric acid (4 mol/L) and ammonium fluoride (15 mol/L). The silicon substrate is etched using 30% KOH at 80 °C. A second BOE etching step is conducted to remove any residual silicon dioxide. The micro-pyramid array mold is thus formed once the oxide layer on the silicon wafer has been completely removed.

A 0.15 wt% dispersion of single-walled CNTs in isopropyl alcohol was purchased from Jiangsu XFNANO Technology Co., Ltd. (Nanjing, China). The dispersion was diluted fivefold with isopropyl alcohol, and 0.05 wt% polyethylene oxide (PEO) was added to suppress the coffee-ring effect during spraying. The solution was then ultrasonicated for 10 min and centrifuged at 500 RPM for 20 min. The upper suspension containing well-dispersed CNTs was collected for spray coating.

To transfer CNTs onto a flexible substrate, a PDMS soft lithography process is used. CNTs are first spray-coated onto a pre-fabricated pyramid array mold. A PDMS mixture with a 1:5 ratio of base-to-curing agent is prepared and degassed using a blender. The mixture is then spin-coated onto the mold at 1000 rpm, yielding a PDMS layer thickness of 25 μm. After spin-coating, the mold is placed in a vacuum chamber to remove residual bubbles, followed by application of a 25 μm PI film as a peeling aid. The sample is then cured in an oven at 120 °C for 20 min. After curing, the resistive sensitive layer is peeled from the mold.

To enable individual signal acquisition in high-density arrays, the TFT active matrix is indispensable for per-pixel addressing. The dual-layer structure integrates a microstructured sensing layer with a 256 × 256 TFT readout array. The TFT array functions as an addressable electrode layer where each transistor gate electron flows through source–drain channels. The active layer of the TFT array is less than 10 μm and the total thickness of the sensing device is less than 100 μm. For texture recognition, the resistive sensitive layer is encapsulated with the TFT chip. Before encapsulation, the chip’s protective film is removed, and PI tape is applied to protect the electrodes. A 10 μm-thick double-sided adhesive is used to fix the sensor in place. Silver paste is applied to the electrode area, and the resistive sensor array is aligned and bonded to the chip. The assembly is then cured on a hot plate at 120 °C for 15 min to ensure stable electrical and mechanical contact. Finally, the flexible printed circuit is bonded using hot pressing and the sensor array is assembled.

In this study, a dual-layer structure is adopted as shown in Figure 1b with a microstructured array as the top sensitive layer and a TFT array as the bottom electrode layer. The electrode density and dimensions are selected for detecting micron-level textures, and the sensitive array is designed accordingly to ensure each TFT pixel is activated under pressure. As shown in Figure 1b, the TFT array features 65,536 pixels, with each size being 85 μm, corresponding to a unit density of 13,840 unit/cm^2^ and an active area of 4.73 cm^2^. Each pixel’s gate controls electron flow by creating or eliminating the channel between the source and drain. The scan line controls switching while the data line selectively read signals, enabling per-pixel data acquisition.

### 2.2. Characterization and Measurements

The sample microstructures were characterized using a field-emission scanning electron microscope (FE-SEM, SU8010, Hitachi, Tokyo, Japan) operated at an accelerating voltage of 5 kV. External pressure was applied and measured using a force gauge (M5-05, MARK-10, Quality Control Solutions, Temecula, CA, USA) with a resolution of 0.5 mN. A computer-controlled three-axis translation stage (PT101(M), Thorlabs, Newton, MA, USA) with 50 nm resolution was used for precise pressure application, driven by DC servo motors and monitored via an integrated displacement sensor. Compression displacement on the microstructures was recorded directly from the stage readings. Electrical resistance was measured using a digital multimeter (34470A, Keysight Technologies, Santa Rosa, CA, USA). 

### 2.3. Finite Element Analysis (FEA) for the Micro-Pyramid Array

The sensor consists of three layers: a middle layer featuring a micro-pyramid array, a flexible PDMS bottom layer, and a rigid glass top layer, as shown in Figure S1. The pyramid array, arranged in a 1 cm^2^ square, is also made of PDMS, chosen for its elasticity. Each pyramid in the array has a base side length b and height H. The glass top layer provides mechanical support and efficient force transmission. A simplified 3 × 3 pyramid array model is used in COMSOL6.2 simulations to study the force–pressure relationship.

A constant force is applied to the upper plate; the relationship between the applied force and the pressure is demonstrated in Equation (1).

A constant force F is applied to the upper plate, producing a pressure *P* ranging from 0 to 4 kPa, as shown in Equation (1). This force is distributed across the sensor array, affecting the deformation of the pyramids and the underlying PDMS layer. To study the impact of structural spacing, pyramid gaps of 10, 20, 30, and 40 μm are analyzed, which are named D10, D20, D30, D40 model, respectively. The focus is on how the contact area A changes with F, as it directly influences sensitivity and texture detection.

(1) $$\;P=\frac{F}{A}$$

Due to non-uniform strain in the 2 μm-thick PDMS substrate, strain must be integrated across the plane when the array is compressed by a displacement △.

(2) $$\;\varepsilon_{avg}=\frac{1}{A_{base}}\iint_{base}\varepsilon (x,y)dxdy$$

where *ε*(*x,y*) represents the local strain, which is related to the pyramid spacing and the distribution of the compressive force. To simplify the calculation, we will only calculate the deformation of a single pyramid under compression, and the strain formula is simplified as follows:

(3) $$\;\varepsilon =\frac{△}{H}$$

According to the stress–strain formula

(4)  σ=E·ε

where *E* is the representation of the elasticity modulus of the micro-pyramids, *σ* and *ε* represent the stress on the micro-pyramids and the strain they generated. According to the similarity law of triangles, the deformation of the pyramid under pressure can be expressed as follows:

(5) $$\;A_{p}={\left(\frac{△}{H}\cdot b\right)}^{2}$$

where *A_p_* stands for the contact area under deformation. The relationship between the reaction force *F_p_* of a single pyramid and the contact area *A_p_* is as follows.

(6) $$\;F_{p}=\sigma \cdot A_{p}$$

By solving Equations (5)–(8) simultaneously, the following equation can be derived:

(7) $$\;F_{p}=\frac{Eb^{3}}{H^{3}}{△}^{3}$$

Therefore, in order to establish the relationship between the simulated contact area and the applied force, the geometric constant *k*_1_ is introduced and defined as

(8) $$\;A_{p}=k_{1}\cdot {F_{p}}^{2/3}$$

By combing the above formula, *k*_1_ can be deduced as

(9) $$\;k_{1}=\frac{b^{2/3}}{E^{2/3}H^{1/3}}$$

Therefore, the contact area *A_P_* is proportional to two-thirds the power of the applied force *F_P_*.

## 3. Results

### 3.1. Characterization of the Sensor Array

**Figure 2:**
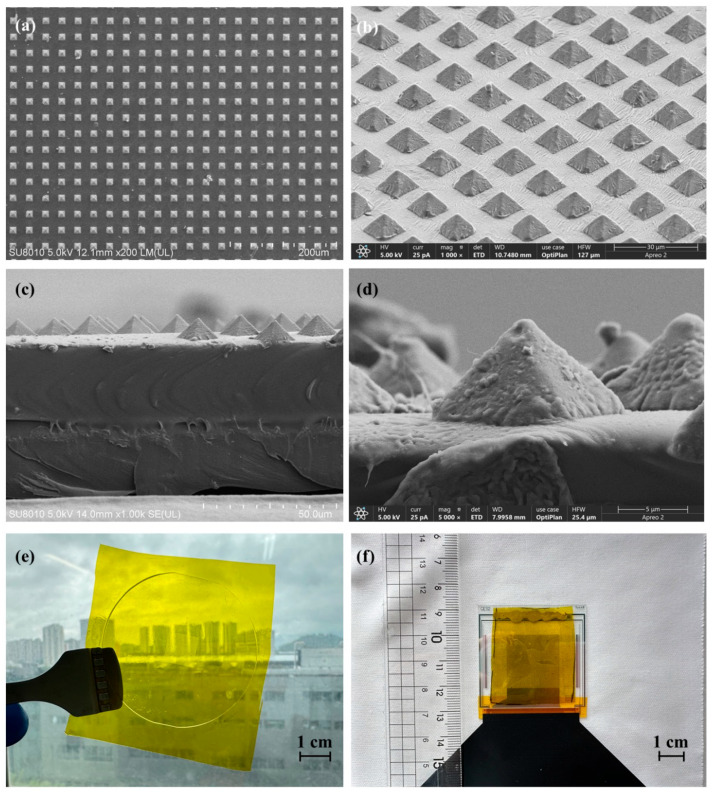
(**a**,**b**,**d**) SEM images of the pyramid array from different angles. (**c**) Cross-sectional view of the resistive sensitive layer. (**e**) Digital image of the resistive sensitive layer. (**f**) Digital image of the sensor device.

Figure 2a is a top view of the high-density pyramid array. The wrinkles observed in Figure 2b,d indicate that the sprayed CNTs are semi-embedded into the PDMS, confirming the successful formation of a flexible sensing layer. Figure 2c shows a cross-sectional view of the resistive sensing layer, where interfacial bonding between the PDMS and the underlying PI layer can be seen, facilitating easier release from the mold. As shown in Figure 2e, the resistive layer exhibits a certain degree of transparency; lower CNT spray density results in higher transmittance but compromises electrical conductivity. Figure 2f exhibits the digital image of the assembled sensor device.

### 3.2. Performance Testing of the Sensor Array

Sensitivity refers to the ratio of the change in output to the change in input when the sensor is in a stable operating state, and is denoted by *S*. The formula is expressed as follows:

(10) $$\;S=\frac{∆I/I_{0}}{∆P}$$

**Figure 3:**
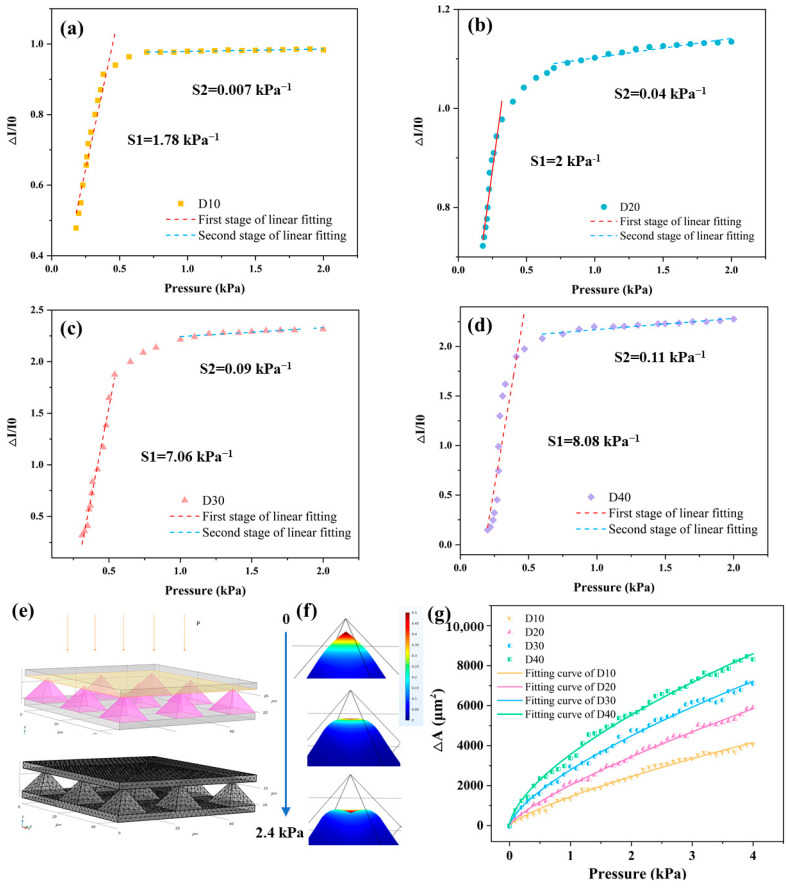
Sensitivity of pyramid sensor array with different spacings. (**a**) 10 μm. (**b**) 20 μm. (**c**) 30 μm. (**d**) 40 μm. (**e**) Simulation model and ultra-fine mesh refinement of the pyramid array. (**f**) Deformation of the model under pressure of 0.6 kPa, 1.2 kPa., 1.8 kPa, and 2.4 kPa, respectively. (**g**) Simulation results showing the sensitivity of pyramid arrays with different spacings.

In Equation (10), ∆*I/I*_0_ represents the change in current, and Δ*P* is the change in pressure. Sensitivity characterizes the sensor’s ability to respond to changes in the input quantity. In this study, sensor arrays with different spacings were tested, and the results are shown in Figure 3. The sensor’s output response is piecewise linear, with sensitivity being higher in the low-pressure range than in the high-pressure range.

The Young’s modulus of PDMS is nonlinear. As compressive strain increases, the Young’s modulus of the material also gradually increases, which reduces the deformation of the sensor under pressure. This nonlinearity in the material’s behavior may explain why the sensor’s pressure response is not entirely linear. At low pressures (0–0.5 kPa), sensitivity is dominated by pyramid-tip deformation, matching simulated contact area trends. Above 0.5 kPa, bulk PDMS compression becomes significant where polymer chain entanglements cause strain hardening. Therefore, the sensitivity curves exhibit two-stage linearity different from the simulation curve. Due to the high array density, a pre-pressure of 0.2 kPa needs to be applied to the sensor array to ensure that all points on the microstructures are in contact with the bottom electrode during the sensitivity test.

By comparing the four figures shown in Figure 3a–d, it is evident that as the spacing between the sensor units increases from 10 μm to 40 μm, the sensitivity S_1_ of the sensor also increases. This trend is consistent with the simulation results, which also demonstrate that increasing the spacing between pyramids enhances the rate of contact area change. The D40 model has the highest sensitivity, which is 8.082 kPa^−1^. Since the pressure required for human hand recognition of sub-millimeter level fine textures is approximately within the range of 0–1.5 kPa, this sensor is capable of meeting the requirements for fine-texture recognition in electronic-skin applications.

To investigate the impact of microstructure density on sensing performance, a Finite Element simulation of the pyramid array is conducted. The pyramid’s geometric configuration, including dimensions and arrangement, directly influences sensor sensitivity and texture detection. A simplified 3 × 3 pyramid array is used in the COMSOL simulation, as shown in Figure 3e. To optimize computational efficiency, a super-refined mesh partitioning strategy (Figure 3f) is applied, ensuring high accuracy in stress-gradient regions while balancing element count to maintain efficiency in the multiphysics simulations.

Figure 3g presents the simulated relationship between contact area and pressure for pyramid arrays with varying spacings. By fitting the results to the theoretical model, a clear correlation is established, validating the model and elucidating the sensor’s mechanical response under pressure. The data further indicate that increasing the spacing between pyramids enhances the rate of contact area change, suggesting improved sensitivity with wider spacing.

**Figure 4:**
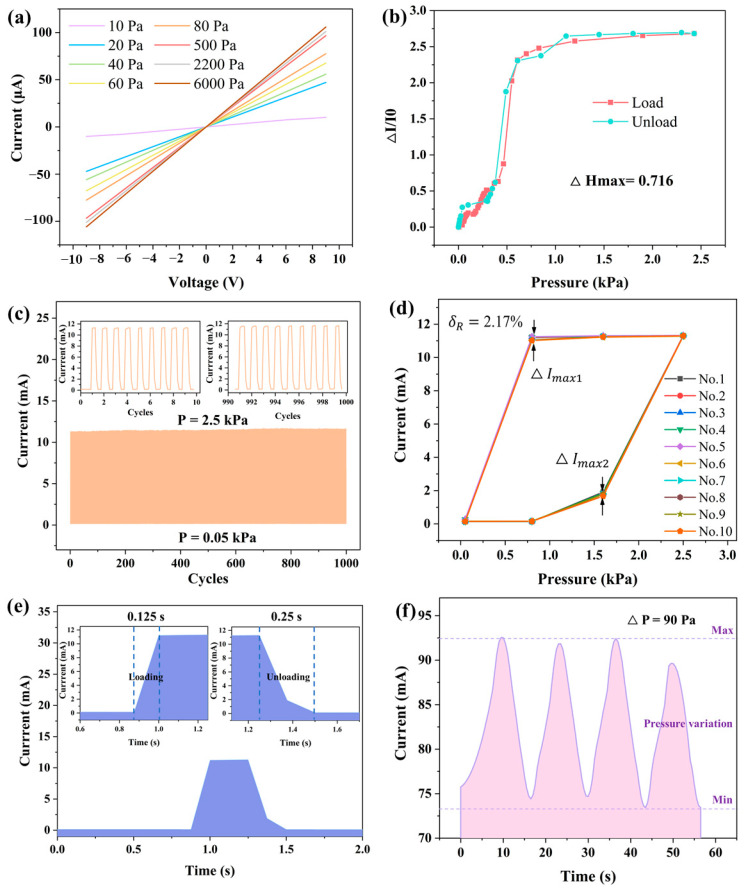
(**a**) Current–voltage curves of the pyramid sensor (0–6 kPa). (**b**) Hysteresis of the pyramid sensor array. (**c**) Durability of the pyramid sensor array loading a pressure of 0.05–2.5 kPa with 1000 cycles. (**d**) Repeatability of the pyramid sensor array. (**e**) Dynamic response speed of the sensor array. (**f**) Minimum resolution of the sensor array.

The sensor’s current–voltage (I–V) characteristics under pressures from 10 Pa to 6 kPa in Figure 4a exhibit a linear response, confirming ideal ohmic contact and stable charge transport. Notably, the significant slope change under 1 kPa indicates high sensitivity to subtle forces, while the symmetric I–V curves demonstrate reliable, reversible performance across the full pressure range.

Hysteresis refers to the degree to which the input–output characteristic curve of a sensor fails to coincide when the input changes from loading and unloading. This phenomenon is commonly represented by $\gamma_{H}$ and can be calculated using Equation (11).

(11) $$\;\gamma_{H}=\pm \frac{{∆H}_{max}}{Y_{FS}}\times 100\%$$

In this context, ${∆H}_{max}$ represents the maximum difference in output between the loading and unloading processes, while $Y_{FS}$ denotes the full-scale output value. The hysteresis of a sensor is primarily influenced by factors such as the materials used and the structural properties of the sensor. In this experiment, it is likely that the viscosity of PDMS plays a significant role in the hysteresis behavior observed. According to Figure 4b, the maximum difference in output is 0.716. Using the calculation from Equation (11), the hysteresis of this sensor is approximately 29.4%. This value indicates how much the sensor’s response differs between the loading and unloading phases, reflecting the influence of material properties on the sensor’s overall performance.

The sensor underwent 1000 fatigue tests between 0.05 and 2.5 kPa, and no significant performance degradation was observed, as demonstrated in Figure 4c. Additionally, as shown in the magnified graphs, the response curves of the sensor during the initial 0–10 cycles and the final 990–1000 cycles are almost identical. Therefore, it can be concluded that the sensor demonstrates good durability and stability, maintaining consistent performance throughout the fatigue testing process.

Repeatability refers to the degree of inconsistency in the output characteristics of a sensor when the input is measured multiple times in the same direction across the full measurement range. In order to quantitatively evaluate the repeatability index of the sensor, the maximum deviation value formula is used for calculation, as shown in the following formula:

(12) $$\;\delta_{R}=\frac{△I_{max}}{Y_{FS}}\times 100\%$$

$Y_{FS}$ refers to the full-scale output of the sensor, $\delta_{R}$ refers to the maximum deviation value, and $△I_{max}$ can be deduced from the following formula:

(13) $$△I_{max}=max(△I_{max1},\;△I_{max2})$$

According to the above formula, the maximum deviation value of the pyramid sensor is calculated to be 2.17% as shown in Figure 4d.

The sensor demonstrates a fast dynamic response, with loading and unloading times of 0.125 s and 0.25 s, respectively (Figure 4e). 

The minimum resolution refers to the sensor’s ability to detect the smallest change in the measured quantity. The sensor can still maintain a stable output under small pressure changes in the range of 230–320 Pa, even with a pressure variation as small as 90 Pa, as shown in Figure 4f. This demonstrates that the sensor is fully capable of operating under low-pressure conditions.

### 3.3. Performance Testing of Individual Sensor Units

To evaluate the uniformity of the sensor array’s response, four sensor units were randomly selected from different locations on the same device, and the rate of current change in response to varying pressure levels was recorded for each. This approach enables a detailed understanding of the performance differences among individual units, ensuring that each one responds consistently during practical use. Such uniformity is critical, as it enhances the overall reliability and stability of the entire sensor array. By analyzing these variations, we can more accurately assess and optimize the sensor’s performance, thereby guaranteeing its excellent functionality in a range of application environments.

**Figure 5:**
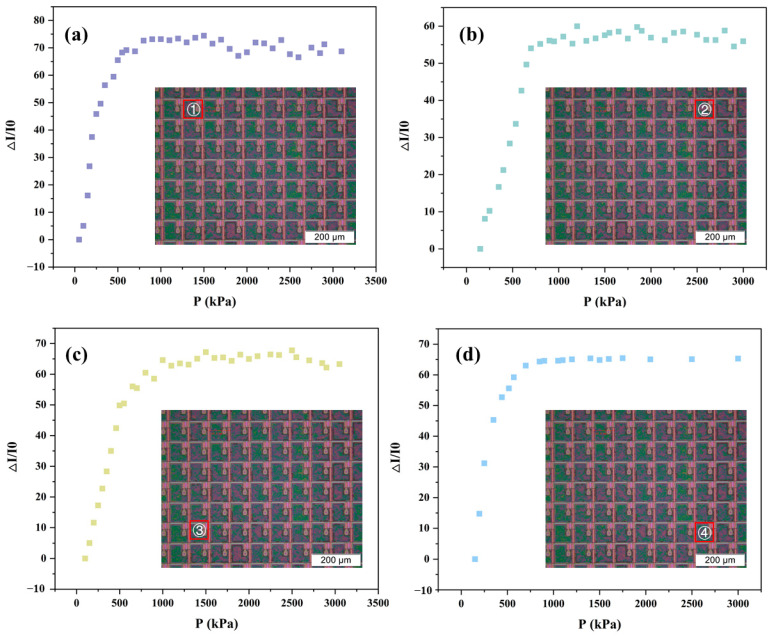
Sensitivity of a single sensing unit on the same pyramid sensor array at different locations. The inserts are the images of the TFT pixels. (**a**) Position No.1. (**b**) Position No.2. (**c**) 3 Position No.3. (**d**) Position No.4.

Figure 5 presents the experimental results, where the response trends of the four sensitive units to pressure variations are largely consistent. Each unit exhibits a two-segment linear trend in its response curve. Due to the relatively lower sensitivity observed in the second segment, our analysis focused solely on the first segment, as determined by the sensitivity calculation formula outlined in Section 3.2. The calculated sensitivities for the four sensor units are 0.126, 0.095, 0.110, and 0.109 kPa^-1^, respectively, with the calculated standard error being 0.634%, indicating that while there are minor variations, the sensor units perform in a generally consistent manner. This low variability suggests good uniformity in the fabrication process and validates the repeatability and reliability of the sensor design. Therefore, the sensor array is well-suited for applications requiring stable multi-point pressure detection.

### 3.4. Texture Recognition of the Sensor Array

**Figure 6:**
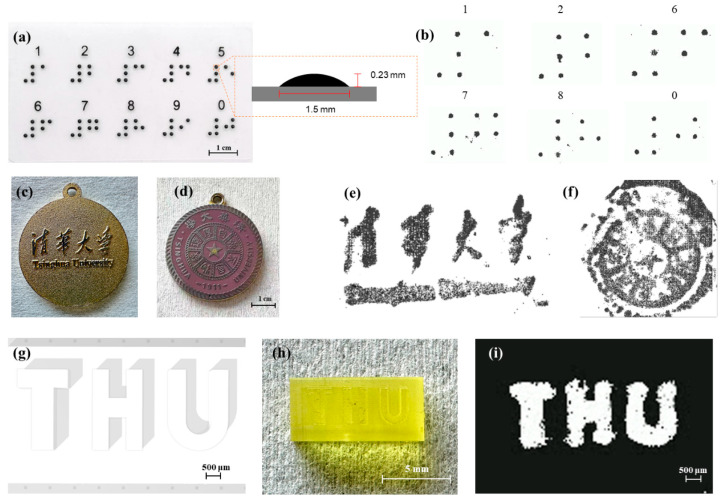
Texture recognition of the sensor with different patterns. (**a**) Braille number board; (**b**) Braille number pattern reconstructed by the sensor; (**c**,**d**) badge of Tsinghua University; (**e**,**f**) Badge pattern reconstructed by the sensor; (**g**,**h**) THU block; (**i**) THU pattern reconstructed by the sensor.

To evaluate the texture recognition capability of the sensor array, pyramid and arrays are tested for Braille recognition. As shown in Figure 6a,b, the sensors clearly resolved Braille dots with a base diameter of ~1.5 mm and a height of 0.23 mm. Subsequently, a Tsinghua University logo is used as a test pattern (Figure 6c,d). Upon pressing the badge onto the array, tactile signals are captured and converted into 2D grayscale images (Figure 6e,f), demonstrating the sensor’s ability to reconstruct large-area textures with overall pattern clarity. However, variations in surface flatness and non-uniform pressure resulted in reduced resolution, indicating the need for further optimization.

Additional testing with THU-patterned textured block which has a minimum line width of 500 µm shows successful reconstruction, as demonstrated in Figure 6g–i.

Although a few reports described high resolution for capacitive and piezoelectric sensors, there are still a substantial manufacturing problem remains, and resolution is still a great challenge in resistive tactile sensors. The performance of present tactile sensor arrays are listed in Table 1, and the comparison confirms our sensor’s superiority in low-pressure sensitivity while maintaining sub-millimeter resolution, addressing a key gap in tactile sensing.

## 4. Conclusions

This work successfully demonstrates a high-density tactile sensor array capable of sub-millimeter texture recognition by integrating pyramid microstructures with a carbon nanotube resistive layer and a 256 × 256 active-matrix TFT readout system. The sensor achieves ultra-high sensitivity (8.082 kPa^−1^ in the 0.2–0.5 kPa range) and 500 μm spatial resolution—surpassing human fingertip discrimination thresholds—while overcoming traditional trade-offs between sensitivity, resolution, and crosstalk. Finite element simulations and experimental validation confirm that optimized pyramid spacing of 40 μm significantly enhances pressure-response characteristics. The device exhibits rapid dynamic response (125 ms loading), exceptional stability (>1000 cycles), and reliable low-pressure detection (down to 90 Pa), with minimal unit-to-unit variability (standard error: 0.634%). Practical validation demonstrates successful reconstruction of 500 μm fine textures, Braille patterns (1.5 mm dots), and complex logos, highlighting its capability for high-fidelity tactile mapping. The scalable fabrication approach, combining mold-based microstructure replication with solution-processable materials, establishes a versatile platform for applications requiring ultra-fine tactile feedback, including advanced prosthetics, robotics, and human–machine interfaces.

## Figures and Tables

**Table 1:** Comparison of performance parameters with other sensors.

Methods	Sensitivity (kPa^−1^)	Range (kPa)	Electrode Density	Spatial Resolution (μm)	Reference
Capacitive	~1.78 × 10^−3^ kPa^−1^(<350 kPa), ~9.65 × 10^−5^ kPa^−1^ (>350 kPa)	35 Pa–330	318 DPI	250	[1]
Piezoresistive	3.1 (0.1–6 kPa), 15.4 (6–18 kPa)	6–18	36 DPI	700	[24]
Piezoelectric	4.4	6.6–3000	1058	20	[27]
Resistive	29.9	50 × 10^−3^–1.06	10 × 10 pixels	5000	[30]
Piezoelectric	/	/	181 DPI	/	[28]
Piezoresistive	3987	0.6–80	80 × 80 pixels	/	[29]
This work	8.082 kPa^−1^ (0.2–0.5 kPa)	0.2–2	460 DPI	500	/

## Data Availability

The data presented in this study are available on request from the corresponding author.

## Supplementary Materials

The following supporting information can be downloaded at: https://www.mdpi.com/article/10.3390/s25165078/s1. Figure S1: Micro-pyramids array model.

## Abbreviations

The following abbreviations are used in this manuscript:

TFT	Thin-film transistor
CNT	Carbon nanotube
BOE	Buffered oxide etch
