# Supplementary material for: High-Density Tactile Sensor Array for Sub-Millimeter Texture Recognition
Source: Sensors (Basel). 2025 Aug 15;25(16):5078. doi: 10.3390/s25165078 (PMC12390072; doi:10.3390/s25165078)
Supplement: Supplementary file 1 [file sensors-25-05078-s001.zip › sensors-3789629-supplementary.pdf]

Supplementary Materials for

High-Density Tactile Sensor Array for Sub-Millimeter Texture Recognition

Chengran Cao [1], Guocheng Wang [1,2] , Yixin Liu [1] and Min Zhang [1,*]

1 Shenzhen International Graduate School, Tsinghua University, Shenzhen 518055, China; ccr22@tsinghua.org.cn (C.C.); wgc22@mails.tsinghua.edu.cn (G.W.); liu-yx19@mails.tsinghua.edu.cn (Y.L.)

2 PengCheng Laboratory, Shenzhen 518000, China

* Correspondence: zhang.min@sz.tsinghua.edu.cn

Figure S1.

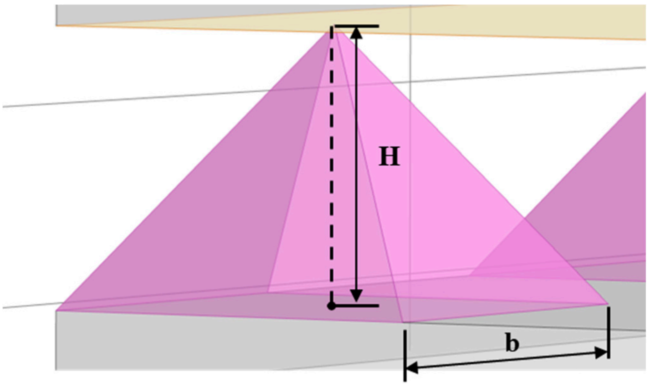

Figure S1. Micro-pyramids array model.
